# Wafer Bonding of SiC-AlN at Room Temperature for All-SiC Capacitive Pressure Sensor

**DOI:** 10.3390/mi10100635

**Published:** 2019-09-23

**Authors:** Fengwen Mu, Yang Xu, Seongbin Shin, Yinghui Wang, Hengyu Xu, Haiping Shang, Yechao Sun, Lei Yue, Tatsurou Tsuyuki, Tadatomo Suga, Weibing Wang, Dapeng Chen

**Affiliations:** 1 Kagami Memorial Research Institute for Materials Science and Technology, Waseda University, Shinjuku, Tokyo 169-0051, Japan; mufengwen123@gmail.com; 2 Collaborative Research Center, Meisei University, Hino-shi, Tokyo 191-8506, Japan; bin912000@naver.com (S.S.);; 3 Institute of Microelectronics of Chinese Academy of Sciences, Beijing 100029, China; xuyang@ime.ac.cn (Y.X.); shanghaiping@ime.ac.cn (H.S.); sunyechao@ime.ac.cn (Y.S.); wangweibing@ime.ac.cn (W.W.); dpchen@ime.ac.cn (D.C.); 4 Kunshan Branch, Institute of Microelectronics of Chinese Academy of Sciences, Suzhou 215347, China; 5 University of Chinese Academy of Sciences, Beijing 100049, China; 6 Institute of Semiconductor & Electronics Technologies, ULVAC, Inc., Susono 410-1231, Japan; lei_yue@ulvac.com (L.Y.); tatsurou_tsuyuki@ulvac.com (T.T.); 7 ULVAC Research Center Suzhou Co., Ltd., Suzhou 215026, China

**Keywords:** all-silicon carbide (SiC), capacitive pressure sensor, room temperature wafer bonding, interface, aluminum nitride (AlN) film

## Abstract

Wafer bonding of a silicon carbide (SiC) diaphragm to a patterned SiC substrate coated with aluminum nitride (AlN) film as an insulating layer is a promising choice to fabricate an all-SiC capacitive pressure sensor. To demonstrate the bonding feasibility, a crystalline AlN film with a root-mean-square (RMS) surface roughness less than ~0.70 nm was deposited on a SiC wafer by a pulsed direct current magnetron sputtering method. Room temperature wafer bonding of SiC-AlN by two surface activated bonding (SAB) methods (standard SAB and modified SAB with Si nano-layer sputtering deposition) was studied. Standard SAB failed in the bonding, while the modified SAB achieved the bonding with a bonding energy of ~1.6 J/m^2^. Both the microstructure and composition of the interface were investigated to understand the bonding mechanisms. Additionally, the surface analyses were employed to confirm the interface investigation. Clear oxidation of the AlN film was found, which is assumed to be the failure reason of direct bonding by standard SAB.

## 1. Introduction

Pressure sensors for harsh environments at high temperature are critical for advanced industrial, automotive, and aerospace sensing applications. Silicon (Si) has been the dominant platform for pressure sensors. However, Si is susceptible to exposure to corrosive mediums and loss of its mechanical reliability at 773 K while Si P-N junctions degrade at temperatures above 473 K, and thus, is not suitable for the pressure sensors in harsh environments with high temperature [1,2,3]. Silicon carbide (SiC) is an excellent candidate material for high-temperature applications because of its wide band-gap, high thermal-mechanical stability, high chemical inertness, and high electrical stability at elevated temperature [3,4,5]. Many researches have employed a SiC pressure sensing diaphragm on a Si substrate [3,4,5,6,7,8,9] but the devices suffer from thermal expansion mismatch between the diaphragm and Si substrate. In recent years, all-SiC pressure sensors have been reported but mainly piezo-resistive types [10,11,12,13]. Piezo-resistive pressure sensors, however, have a small gage factor and a significant temperature-dependence as well as suffer from contact resistance variation at elevated temperature, which would bring an inferior sensor performance. Capacitive pressure sensors can overcome the stated disadvantages of piezo-resistive pressure sensors, already realized for wireless sensing schemes [9,14]. However, reports of all-SiC capacitive pressure sensors are very few. Only L. Chen et al. published an all-SiC capacitive pressure sensor, which is comprised of a SiC diaphragm on a poly SiC substrate. The SiC diaphragm was deposited by low pressure chemical vapor deposition and released by a chemical etching process [14].

**Figure 1:**
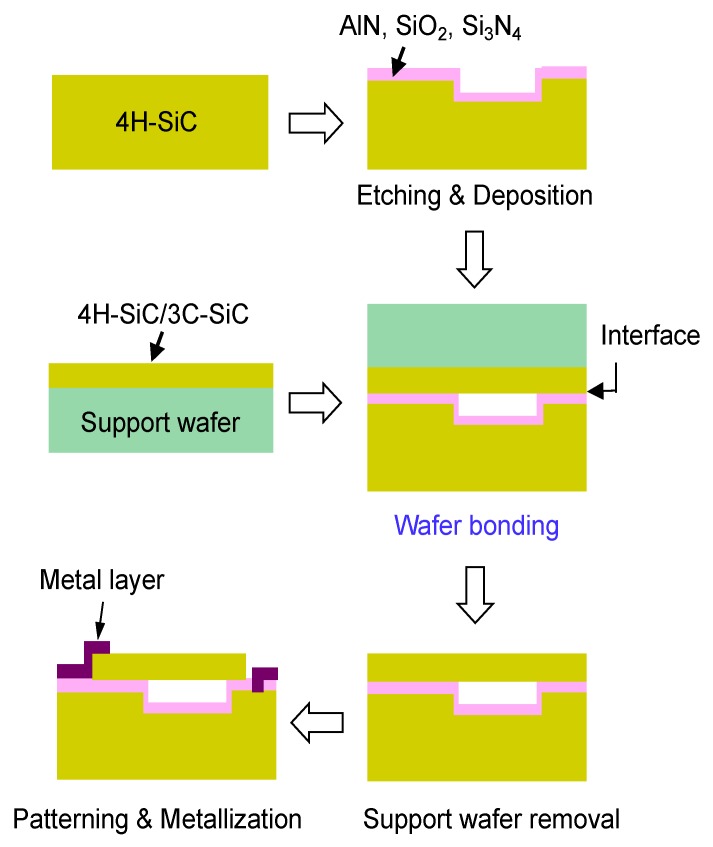
A simplified schematic process to fabricate the all-silicon carbide (SiC) capacitive pressure sensor via wafer bonding.

SiC-diaphragm bonding onto a patterned SiC substrate coated with an insulating film is another choice to fabricate an all-SiC capacitive pressure sensor, in which the real bonding is between SiC and the insulating layer. The SiC-diaphragm wafer could be a 3C-SiC epi-layer grown on a Si substrate or a SiC layer transferred on a support substrate. A simplified schematic illustration of the above proposal is shown in Figure 1. For the insulating layer between diaphragm and substrate, there are several choices such as Si_3_N_4_, SiO_2_, and AlN. Compared to Si_3_N_4_ and SiO_2_, AlN shows a long-term high-temperature stability and a similar thermal expansion coefficient as that of SiC, which means the reduction of error and failure owing to the small thermal expansion mismatch between diaphragm and substrate [15,16,17,18].

To our best knowledge, the bonding of SiC to AlN is hardly reported; thus, it is worth to demonstrate the wafer bonding of SiC to AlN. In this study, an AlN film is deposited on a SiC substrate for the bonding demonstration of SiC-AlN. A second SiC wafer, same as that for AlN deposition, is used as the SiC-diaphragm wafer. Prior to bonding, the deposited AlN film is inspected by dynamic force microscopy (DFM) and scanning electron microscopy (SEM). After bonding, the bonded wafer is characterized in terms of bonding void and bonding energy. Further, the bonding mechanisms are investigated through interfacial analyses using scanning transmission electron microscopy (STEM) and electron energy loss spectroscopy (EELS). In addition, surface analysis using X-ray photoelectron spectroscopy (XPS) is carried out to confirm the interfacial analyses.

## 2. Materials and Methods 

A ~100 nm-thick AlN film was deposited on the Si-face of a commercial 4H-SiC substrate (4-inch, ~355-μm-thick, n-type, 4° off towards [11,12,13,14,15,16,17,18,19,20]) by a pulsed direct current magnetron sputtering machine (ULVAC SME-200) for bonding demonstration. Pure N_2_ gas was used for the sputtering and the background vacuum of the sputtering chamber was 1 × 10^−5^ Pa. The Si-face used for deposition was smoothed by chemical mechanical polishing (CMP) to achieve an RMS surface roughness of ~0.3 nm. Before the AlN deposition in wafer size, an optimization process to reduce the surface roughness was conducted in chip size (10 mm × 10 mm) by controlling the substrate temperature. In the temperature range from 573 K to 973 K, the higher the temperature, the smaller surface roughness. Therefore, the deposition for bonding was carried out at a substrate temperature of 973 K to get a smooth surface, which is critical to get a successful bonding at a low temperature. After deposition, the surface roughness and microstructure of the AlN film were investigated by DFM and SEM. Here, a second 3-inch SiC wafer, same as that for deposition, was used as the SiC-diaphragm wafer for the bonding demonstration.

Surface activated bonding method was employed for bonding of SiC to AlN at room temperature, which could avoid any thermal damages or residual stress caused by thermal expansion mismatch when the real thin SiC film would be used. Both standard SAB and modified SAB with Si nano-layer sputtering deposition were applied to realize the bonding of SiC-AlN. Since Si-Si bonding by SAB can be as strong as bulk material [19], the Si nano-layer in modified SAB is expected to enhance the bonding. The standard SAB process consists of two steps: firstly, the wafer surfaces are activated by the Ar ion beam irradiation with the accelerating voltage and current of 1 kV and 100 mA, respectively; secondly, after surface activation, the two activated wafers are contacted directly under ~1.0 MPa for 300 s at room temperature in ultra-high vacuum (UHV). The modified SAB with Si nano-layer sputtering deposition contains four steps: the first is also surface activation but followed by Si sputtering deposition via Ar ion beam on both wafers; then surface activation of the Si deposited layers is done; finally, the bonding is conducted under ~1.0 MPa for 300 s in UHV. The accelerating voltage of the ion beam source for both surface activation and sputtering deposition is 1 kV, and the currents of the ion beam source for surface activation and sputtering deposition are 200 mA and 400 mA, respectively. After bonding, the bonded wafer was characterized in terms of bonding void and bonding energy by scanning acoustic microscope (SAM) with a pixel size of 50 μm and by crack-opening method. In the crack-opening method [20,21], a razor blade is inserted to measure the bonding energy (*γ*) in air at room temperature, which is the fracture energy of bonding interface. Since the thickness of the AlN layer could be neglected compared with that of the SiC substrate, the two wafers are considered as same substrate. Therefore, the bonding energy is calculated by the following equation [20]:

(1) $$\gamma =\frac{{3t}_{b}^{2}Et_{w}^{3}}{{32L}^{4}}$$

where E = 530 GPa is selected as the Young’s modulus of single crystalline 4H-SiC [22], *t*_w_ is the thickness of wafer, *t*_b_ is the thickness of blade, and *L* is the crack length. To understand the bonding mechanism, the microstructure and composition of the bonding interface were investigated by STEM and EELS. To confirm the results of STEM and EELS, different sample surfaces were further analyzed by XPS with an Al X-ray source (1486.6 eV).

## 3. Results and Discussion

**Figure 2:**
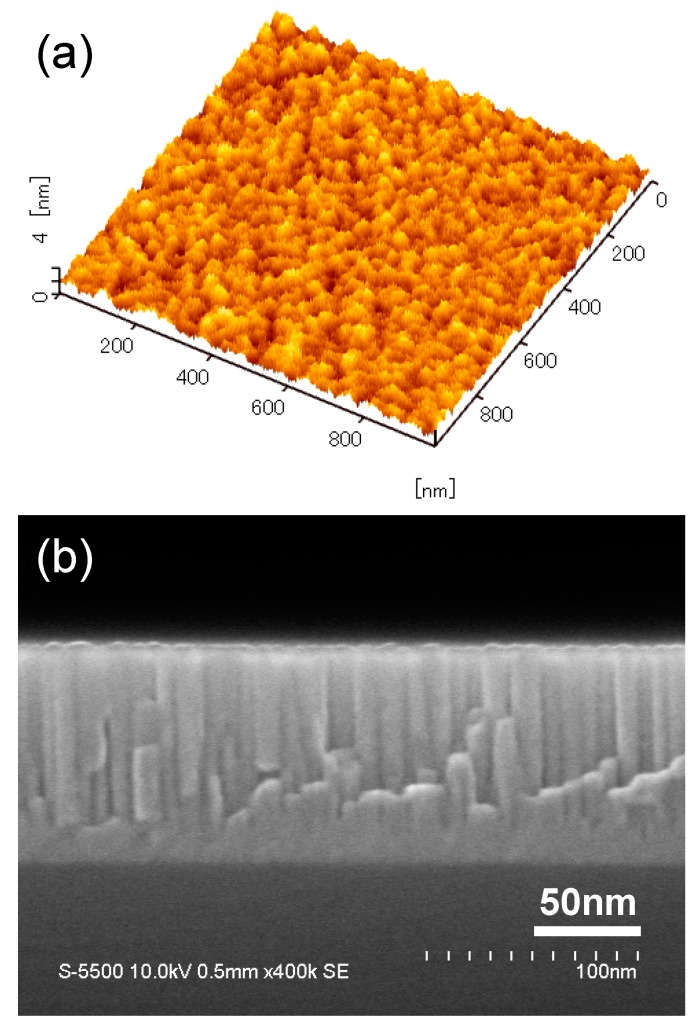
(**a**) Dynamic force microscopy (DFM) image of the surface (1μm × 1μm) of aluminum nitride (AlN) film (in chip size) deposited at a substrate temperature of 973 K and (**b**) cross-sectional scanning electron microscopy (SEM) image of the AlN layer deposited on SiC.

The RMS surface roughness of the AlN layer deposited on a SiC chip at temperature of 973 K can be ~0.55 nm, as shown in Figure 2a. When the AlN deposition is conducted in wafer size, the wafer center can have a similar RMS surface roughness as in chip size while that of wafer edge areas is ~0.64 nm. Figure 2b shows the cross-sectional SEM image of the AlN on SiC. The film has a uniform thickness of about 100 nm. Additionally, the AlN layer consists of numerous highly-oriented columnar grains, perpendicular to the surface of SiC substrate, which are very likely along the *c*-axis (0002) orientation. Similar structure has been found in previous researches [23,24,25].

**Figure 3:**
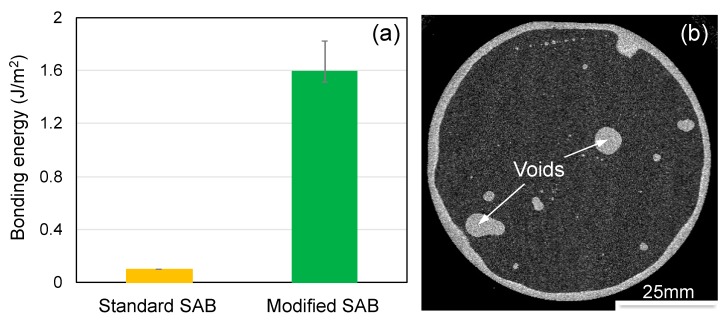
(**a**) Comparison of bonding energy of SiC-AlN using standard surface activated bonding (SAB) and modified SAB with Si nano-layer sputtering deposition and (**b**) scanning acoustic microscope (SAM) image of the bonded wafer of SiC-AlN.

The bonding energy of SiC-AlN by standard SAB and modified SAB with Si nano-layer sputtering deposition is compared in Figure 3a. The bonding of SiC to AlN by standard SAB fails, while modified SAB can achieve a successful bonding with a bonding energy of ~1.6 J/m^2^. The SAM image of the bonded wafer by modified SAB is illustrated in Figure 3b, which shows that most of the wafer is bonded except some voids and the edge exclusion area. The ratios of the voids area and the edge exclusion area are ~5% and ~10%, respectively. According to our experiences, the voids are formed by particle contaminations since the bonding experiments were not carried out in a clean room. Experimental operation in cleanroom can reduce the voids area.

**Figure 4:**
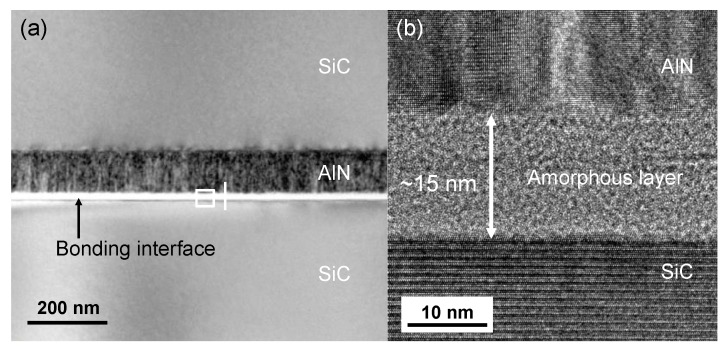
(**a**) Scanning transmission electron microscopy (STEM) image of the bonded SiC-AlN interface at a low magnification and (**b**) the magnification of the rectangular area in (**a**).

**Figure 5:**
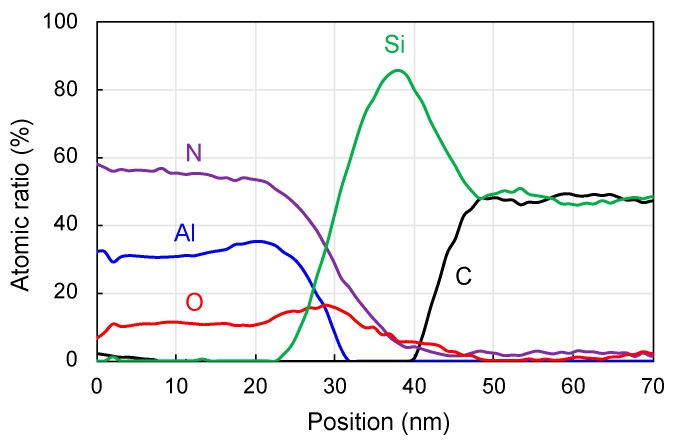
Electron energy loss spectroscopy (EELS) scanning along the white line in Figure 4a. The profiles of Si, C, Al, N, and O are represented by green, black, blue, purple, and red.

To understand the bonding mechanisms and the failure of standard SAB, STEM and EELS were employed to analyze the microstructure and composition of the bonding interface and the AlN surface layer. Figure 4a shows the structure of SiC-AlN-SiC. No clear voids or cracks are seen. The thickness of the deposited AlN layer is in agreement with the measurements in the SEM examination mentioned above. The rectangular area in Figure 4a is further magnified to show the interfacial structure, as displayed in Figure 4b. There is a seamless amorphous layer with a thickness of ~15 nm at the bonding interface of SiC-AlN. According to our previous researches [26,27], the surface activation of ion beam bombardment will cause the formation of amorphous layer. Therefore, the amorphous layer may consist of amorphous SiC, amorphous AlN, and deposited Si layer. The total thickness of the two deposited Si layers is around 10nm according to our previous measurement. Besides, lattice structure can be observed on the AlN side, which confirms that the deposited AlN has a crystalline structure. The result of EELS scanning along the white line in Figure 4a is shown in Figure 5. The profiles of Si, C, Al, N, and O are represented by green, black, blue, purple, and red. The interfacial Si layer can be clearly identified, and the deposited AlN film is N-enriched to some extent. The surface layer of AlN after surface activation seems still N-enriched. It is noteworthy that O is detected in both of the surface layer and the inside of deposited AlN film, which indicates the oxidization of AlN during deposition. This oxidation may be the cause of the failure of the direct bonding with standard SAB. This is in agreement with earlier observations of the weak bonding of SiC to oxide using standard SAB [28].

**Figure 6:**
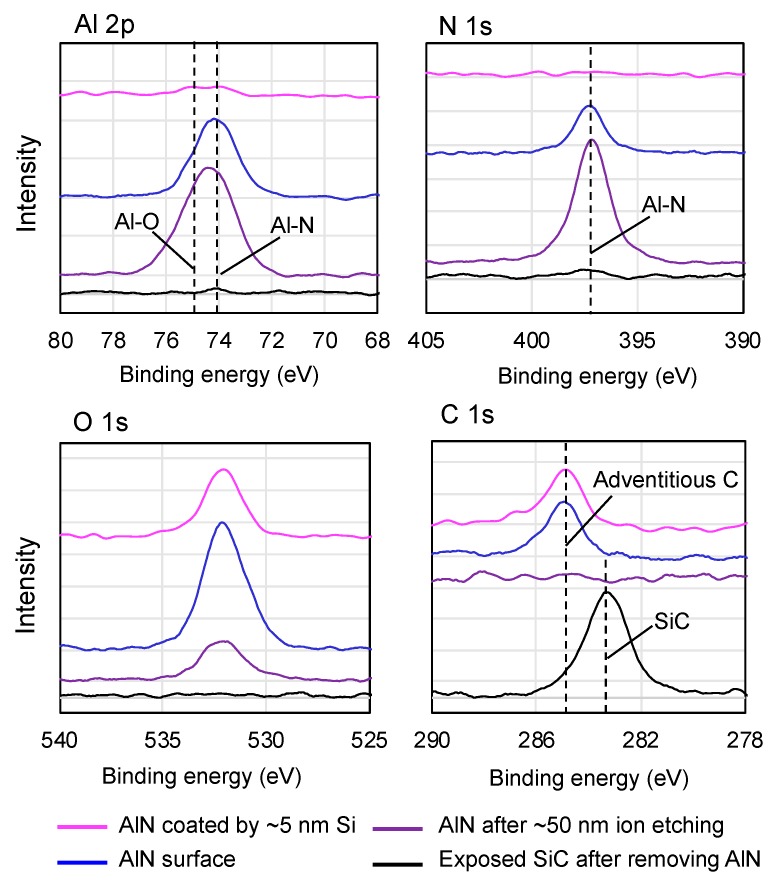
X-ray photoelectron spectroscopy (XPS) spectra of Al 2p, N 1s, O 1s, and C 1s taken from AlN film coated with ~5 nm Si layer (used for bonding in modified SAB), AlN film, the AlN film after ~50 nm etching by ion beam, and exposed SiC substrate after the complete etching of AlN, which are highlighted by magenta, blue, purple, and black.

To confirm the uniformity of deposited Si layer and the oxidation of AlN film, XPS spectra, as shown in Figure 6, are taken from four different surfaces: AlN film coated with ~5 nm Si layer (used for bonding in modified SAB), AlN film, AlN film after ~50 nm etching by ion beam equipped in XPS analysis chamber, and exposed SiC substrate after the complete etching of AlN film. The C 1s peak position of the adventitious carbon at 284.8 eV is used as the charge reference. According to the XPS spectra taken from the AlN film coated by ~5 nm Si layer, no Al can be detected from the AlN layer, which indicates the uniform deposition of Si layer. By comparison of the XPS results of both the AlN film and the AlN film after ~50 nm etching, the oxidation can be clearly confirmed from O 1s spectra. Moreover, their Al 2p peak positions have visible difference. The Al 2p peak of the AlN film locates at ~74.1 eV, agreeing with the published data [29], while the peak of the etched AlN film is shifted towards the peak of Al-O at ~75.0 eV [30]. This further confirms the oxidation of AlN during deposition, and the internal AlN seems more seriously oxidized. Very interestingly, both N 1s peaks of the two samples are at ~397.0 eV with a symmetrical shape, which implies they are likely from AlN [29]. Besides, no N-O peak is found in the N 1s spectra [31], which does not exhibit the existence of N-O. One possible O source might be the inside of SiC substrate; however, the spectra of the SiC after complete etching of AlN film confirms that there is no O inside SiC. The relative low background vacuum of the chamber may cause the oxidation of AlN film during deposition at a high temperature. This may be avoided by the improvement of the background vacuum of the chamber in the future.

## 4. Conclusions

Wafer bonding of SiC-AlN for the fabrication of all-SiC capacitive pressure sensor is proposed and demonstrated in this study. For bonding demonstration, a crystalline AlN film with a thickness of ~100 nm and an RMS surface roughness less than ~0.7 nm was deposited on a SiC wafer by a pulsed direct current magnetron sputtering method at a substrate temperature of 973 K. Both standard SAB and modified SAB with Si nano-layer sputtering deposition were applied to the SiC-AlN bonding at room temperature. Standard SAB failed in the bonding, while the bonding using modified SAB had a bonding energy of ~1.6 J/m^2^, which is strong enough to withstand common mechanical cutting process [27]. The microstructure and composition of the bonding interface were investigated by STEM and EELS. A ~15 nm amorphous layer was observed at the interface, which consisted of amorphous SiC, amorphous AlN and deposited Si nano-layer. The N-enrichment of AlN film was confirmed, which can explain why the surface layer of AlN after surface activation seemed N-enriched. Notably, the oxidation was detected in the AlN side. Surface analyses by XPS further confirmed the AlN oxidation and the uniformity of the deposited Si nano-layer in the modified SAB method. The AlN oxidation was considered to be a possible failure reason of the direct bonding with standard SAB.
